# Health Tract, No. 145

**Published:** 1865

**Authors:** 


### RESIGNATION.

ONE of the most instructive articles we have read for a long time on the true meaning, nature, and uses of "*resignation*," is found in the *Atlantic Monthly* for April, 1863. It is full of a sound philosophy, and we certainly urge our readers, whether old or young, sick or well, fortunate or unfortunate, if they can possibly save twenty-five cents, to procure the number and read and study, and read it again from beginning to end. We have felt the truth of its sentiments a thousand times as a physician. It is said that there is but a step from the sublime to the ridiculous; it is just as true that there is but a step between courage and cowardice in this matter of "resignation;" but that step is the distance between life and death to many an invalid. One man is sick, and laying the blame of it on the Almighty, whines out, "It's the Lord's will," and sits about and lounges and loafs around for weeks and months, waiting to get well. We verily believe that full one half of such people, if not all of them, don't want to get well, for then they would have to get up and do something. There is another class, true men and women, persons of force are they, and capable of great deeds, who shake off sickness, and sloth, and idleness, and a craven submission to the mishaps which may befall them; believing fully that resignation is a grace only when it bows to what can not be helped, and was not brought on by wickedness or the want of wisdom on their part. If calamities come upon us without our fault, and at the same time are clearly beyond removal by any power of our own, then a dignified and submissive resignation is a nobility, which only a great heart can achieve; then there is a sweetness in resignation which pays for all that it cost; for, while bending the knee and bowing the head, the eye looks trustingly upward, and, piercing through the black and threatening cloud, discerns the gladdening sun in the distance, and patiently and piously bides its time. This is that faith in God which sanctifies and raises man to be akin to angels. If a man fails in business, it is not at any time of life a true resignation to give up for the remainder of his days and make no further effort to recover himself, any more than it is a true resignation for a man who gets sick to cry out, "The will of the Lord be done," as if it could be his will to see a child of his suffer,

"For we his offspring are."

He may permit suffering, but he has no agency in bringing it on any creature of his. As long as sickness and trouble are the results of our own wrong-doing, of our yielding to sense and passion and appetite, instead of abandoning ourselves to helplessness under the deceitful plea of a pious resignation, we should heroically shake them off as a viper or as some deadly spell. The mishaps of life are the result of ignorance, carelessness, or wickedness of ourselves or others; we should in every case seek out the specific cause, and if in our ourselves, rectify it, if from the misdoing of others, endeavor to rectify it also; and if no human efforts can accomplish such a rectification, then, and not till then, is it a true heroism and a sterling piety, a genuine "resignation," to say in loving confidence and hope: "THY WILL BE DONE!"

